# A cluster of blood-based protein biomarkers associated with decreased cerebral blood flow relates to future cardiovascular events in patients with cardiovascular disease

**DOI:** 10.1177/0271678X231195243

**Published:** 2023-08-12

**Authors:** L Malin Overmars, Sanne Kuipers, Bram van Es, Jeroen de Bresser, Esther E Bron, Imo E Hoefer, Wouter W Van Solinge, L Jaap Kappelle, Matthias JP van Osch, Charlotte E Teunissen, Geert Jan Biessels, Saskia Haitjema, Heart-Brain Connection Consortium

**Affiliations:** 1 Central Diagnostic Laboratory, University Medical Center Utrecht, Utrecht University, Utrecht, the Netherlands; 2 Department of Neurology, UMC Utrecht Brain Center, University Medical Center, Utrecht University, Utrecht, the Netherlands; 3 MedxAI, Theophile de Bockstraat 77-1, Amsterdam, the Netherlands; 4 Department of Radiology, Leiden University Medical Center, Leiden, the Netherlands; 5 Department of Radiology & Nuclear Medicine, Erasmus MC, Rotterdam, the Netherlands; 6 Neurochemistry Laboratory, Department of Clinical Chemistry, Amsterdam Neuroscience, Amsterdam UMC, Vrije Universiteit Amsterdam, Amsterdam, the Netherlands

**Keywords:** Cardiovascular diseases, cerebral blood flow, heart-brain connection, hemodynamics, proteomics

## Abstract

Biological processes underlying decreased cerebral blood flow (CBF) in patients with cardiovascular disease (CVD) are largely unknown. We hypothesized that identification of protein clusters associated with lower CBF in patients with CVD may explain underlying processes. In 428 participants (74% cardiovascular diseases; 26% reference participants) from the Heart-Brain Connection Study, we assessed the relationship between 92 plasma proteins from the Olink® cardiovascular III panel and normal-appearing grey matter CBF, using affinity propagation and hierarchical clustering algorithms, and generated a Biomarker Compound Score (BCS). The BCS was related to cardiovascular risk and observed cardiovascular events within 2-year follow-up using Spearman correlation and logistic regression. Thirteen proteins were associated with CBF (ρ_Spearman_ range: −0.10 to −0.19, p_FDR-corrected_ <0.05), and formed one cluster. The cluster primarily reflected extracellular matrix organization processes. The BCS was higher in patients with CVD compared to reference participants (p_FDR-corrected_ <0.05) and was associated with cardiovascular risk (ρ_Spearman_ 0.42, p < 0.001) and cardiovascular events (OR 2.05, p < 0.01). In conclusion, we identified a cluster of plasma proteins related to CBF, reflecting extracellular matrix organization processes, that is also related to future cardiovascular events in patients with CVD, representing potential targets to preserve CBF and mitigate cardiovascular risk in patients with CVD.

## Introduction

Patients with cardiovascular disease (CVD) are at risk for structural and functional brain damage, likely involving cerebral hemodynamic disturbances, including a gradual decline in cerebral blood flow (CBF).^1^ Within the Heart Brain Connection (HBC) study we explore the role of hemodynamic disturbances in patients with CVD by relating CBF to vascular brain injury and cognitive functioning in three exemplar conditions for hemodynamic impairment at different levels of the heart-brain axis,^2^ namely heart failure (HF), carotid occlusive disease (COD), and vascular cognitive impairment (VCI).

Studying factors underlying cellular and molecular processes affecting CBF may provide leads on the biological mechanisms of the adverse consequences of CVD on the brain. For example, endothelial dysfunction was previously suggested as a possible contributing factor to reduced CBF in the marginally perfused white matter.^3^ Also, oxidative stress and inflammation processes have been related to CBF.^3^ However, the exact biological processes underlying decreased CBF in CVD are largely unknown.

Biological processes that are involved in decreased CBF may be reflected in levels of circulating blood-based protein biomarkers. Even though recent studies have examined single proteins related to reduced CBF, e.g. matrix metalloproteinase-2 (MMP2) originating from the microglia and the endothelium based on a rodent model,^4^ no comprehensive protein studies with large panels have yet been done in this context. To identify biological processes involved, interactions between circulating proteins should be considered. This could be done by addressing them in a cluster-based way,^5–7^ since several proteins can be involved in the same biological process and one protein can be involved in several different biological processes. Exploring relations of multiple proteins with decreased CBF simultaneously may provide a broader perspective on mechanisms at play.

Therefore, to provide leads on biological mechanisms affecting CBF in patients with CVD, we evaluated protein biomarkers from the Olink® cardiovascular III panel^8^ of participants from the HBC study. This panel comprises 92 proteins which were selected in collaboration with experts from the cardiovascular field. It contains proteins that are known to be related to cardiovascular risk (e.g. GDF-15 and ST-2^9–11^) as well as some exploratory proteins with potential as new cardiovascular markers. We hypothesized that clustering of proteins in relation to CBF and to each other offers indications on key biological processes involved in decreased CBF in CVD. We tested this hypothesis in patients with CVD with hemodynamic disturbance at different levels of the heart-brain axis and reference participants, by exploring relationships of proteins from the panel with decreased CBF, as well as relationships between proteins based on prior knowledge using data-driven cluster analyses. Additionally, to test the clinical relevance of our findings, we compared a biomarker compound score (BCS) derived from the plasma protein cluster between participant groups and explored how the BCS related to cardiovascular risk and observed cardiovascular events within two-year follow-up.

## Methods

### Study population

We tested our hypothesis with retrospective analysis in a prospectively enrolled cohort of individuals who participated in the HBC study, a study with a follow-up measurement after two years. The HBC study includes patients with different manifestations of CVD with hemodynamic disturbance at different levels of the heart-brain axis, including heart failure (HF), carotid occlusive disease (COD), and vascular cognitive impairment (VCI), and a reference group. Participants were recruited from cardiology, memory, and neurology outpatient clinics in four university medical centers in the Netherlands. Reference participants were recruited via advertising leaflets and among spouses of participants. The rationale, design, and inclusion criteria of the HBC study have been described elsewhere.^2^ For the current study, we included participants from the HBC study who had both available CBF measurements (3T pseudo-continuous arterial spin labeling (pCASL) on magnetic resonance imaging (MRI)), and an available cardiovascular protein biomarker panel at the baseline visit. We excluded participants with pCASL scans of suboptimal quality (i.e., incomplete pCASL-sequence, artefacts) (n = 111). All participants provided written informed consent. The Medical Ethics Review Committee of the Leiden University Medical Center provided central approval. Local medical ethical committees of all sites approved the local performance of the study. The Heart-Brain Study is performed in accordance with the declaration of Helsinki (version 2013) and the Dutch Medical Research Involving Human Subjects Act (WMO).

### MRI protocol and preprocessing

Brain MRIs were acquired on Philips Ingenia, Achieva and Gemini 3 T MRI scanners.^2^ Normal appearing gray matter CBF was measured with pCASL (multi-slice 2D echo-planar imaging [EPI] acquisition with background suppression; labeling duration = 1800 milliseconds; post-labeling delay = 1800 milliseconds; single-shot EPI readout; resolution = 3 × 3 ×7 mm^3^).^2,12^ pCASL data were processed using the automated Iris pipeline for CBF quantification.^13^ Quantification of pCASL data into CBF maps was based on a single- compartment model after the subtraction of labeled images from control images.^12^ To scale the signal intensities of the subtracted pCASL images to absolute CBF units, a separately acquired proton density weighted image was used. The quantification further included motion-correction of the raw pCASL data^14^ and partial volume correction (PVC). CBF was quantified in normal-appearing gray matter only. To obtain the normal-appearing gray matter mask for each participant, first a binary gray matter segmentation was obtained. Subsequently, PVC-uncorrected pCASL images of all participants were visually inspected.^12^ Images with suboptimal quality (i.e., motion artefacts, incomplete ASL-sequence, or labeling errors) and images with dominant vascular artefacts and little tissue perfusion signal were not used for further analyses.^15^ As a sensitivity check of the pCASL data we additionally used phase contrast flow measures divided by total brain volume (in ml/100 g/min) for total CBF (methods have been described previously in more detail).^16^

### Definition of clinical characteristics

Clinical characteristics and vascular risk factors were registered by trained physicians or research nurses using a standardized interview and physical examination. Hypertension was defined as the use of antihypertensive drugs, as systolic blood pressure ≥140 mm Hg, a diastolic blood pressure ≥90 mm Hg, or the presence in medical history. Obesity was defined as a body mass index of ≥30. Diabetes, previous stroke, and previous myocardial infarction were defined as presence in medical history.

We defined cardiovascular risk based on the validated and integrated Systematic COronary Risk Evaluation (SCORE),^17^ defined as a composite of sex; age; total cholesterol; systolic blood pressure; and smoking status. We defined cardiovascular events as a composite of cardiac death; myocardial infarction; stroke; and terminal heart failure within 2-year follow-up.

### Assessment of blood-based cardiovascular biomarkers

Participants provided blood samples which were collected into ethylenediaminetetraacetic acid (EDTA) plasma vacutainer tubes at the same day as the assessment of the clinical characteristics. Cardiovascular protein biomarker values were measured in whole blood samples with a multiplex immunoassay using the Olink® Proteomics Cardiovascular III panel.^8^ This panel comprises 92 CVD-related protein biomarkers, selected by experts from the cardiovascular field. Raw biomarker values were converted to Olink®'s arbitrary unit, Normalized Protein eXpression (NPX), a relative unit on a log2-scale. The proteins included in the Olink® Cardiovascular III panel can be found in Supplemental Table S1.

### Statistical analysis

To identify indications on key biological processes involved in decreased CBF in CVD, we conducted a comprehensive step-by-step analysis.

#### Step I - Confirm relationships between CBF, CVD, and cardiovascular risk

CBF and cardiovascular risk (SCORE) in the four participant groups were compared using a Welch’s one-way ANOVA test with Games-Howell post-hoc tests. A p_FDR-corrected_ < 0.05 was considered statistically significant. The association between CBF and cardiovascular risk (SCORE) was assessed with a Spearman’s correlation analysis, p < 0.05 was considered statistically significant.

#### Step II – Identify cluster of biomarkers and reflected biological processes associated with CBF

To identify biological mechanisms involved in the pathophysiology of reduced CBF, we applied a modified version of a previously published cluster-based methodology for data-driven prioritization of biomarkers and adapted it to the current research question.^5,7^ First, we identified associations between each of the 92 cardiovascular proteins involved in the Olink cardiovascular III panel and CBF with Spearman’s correlation analyses. Given the exploratory nature of this research, we aimed to reduce the risk of false negatives. Therefore, we chose an FDR-correction for multiple testing rather than a more conservative Bonferroni p-value correction. P_FDR-corrected_ < 0.05 were considered significant. The thus identified proteins were then analyzed in a cluster-based way, both using prior biological knowledge on interactions between proteins captured in the STRING database,^18^ as well as in an unsupervised data-driven way, using affinity propagation and agglomerative clustering algorithms based on the Olink® NPX values.^19^

In STRING,^18^ the names of the proteins that correlated significantly with CBF were entered in the ‘Multiple proteins’ section of the tool. Next, we used the affinity propagation algorithm to identify the optimal number of clusters, after which we performed agglomerative clustering based on the Pearson’s correlation matrix, which was constructed based on the Olink® NPX values. Since the choice of number of clusters in agglomerative clustering is arbitrary, we used the affinity propagation algorithm to substantiate the choice of the number of clusters, as mentioned above. If a biomarker was clustered together with the proteins involved in the STRING cluster(s) in 500 bootstrapped subsample replicates of the data, we added this biomarker to the cluster(s). Of note, several biomedical factors (such as age and sex) might be associated with both CBF and blood-based biomarkers. Adding these biomedical factors to the analyses as potential confounders might lead to overadjustment, if a biomarker would be in a causal path between a factor and the outcome CBF. Hence, in this first step we used unadjusted analyses.

Second, to give biological meaning to the biomarker cluster(s) by identifying reflected biological processes, we performed a pathway analysis using the Reactome pathway analysis tool.^20^ Reactome uses Fisher’s exact test to identify enriched pathways within the cluster(s).^20^ Pathways with p < 0.05 and p_FDR-corrected_ < 0.05 were considered statistically significant enriched.

Third, to create a variable that captures the values of the biomarker cluster(s) in one score per cluster for further analyses, we calculated a Biomarker Compound Score (BCS). The BCS was constructed by minimizing the summed L2-error with the individual biomarkers. A differential evolution algorithm ^21^ from the Python SciPy library^22^ was used for this purpose.

We performed a Spearman correlation analysis to test the association between the BCS and CBF across and within participant groups (HF, COD, VCI and reference participants).

#### Step III - Determine the association between the BCS, cardiovascular risk and cardiovascular events

The BCS was compared between the four participant groups using a Welch’s one-way ANOVA test with Games-Howell post-hoc tests. p_FDR-corrected_ < 0.05 was considered statistically significant. Additionally, we tested the association between the BCS and cardiovascular risk (SCORE) with a Spearman’s correlation analysis, p < 0.05 was considered statistically significant. Lastly, we tested the association between CBF and BCS and cardiovascular events across all participants, adjusted for cardiovascular risk (SCORE), using logistic regression analyses.

Shapiro-Wilk tests were performed to test for normality. The ggstatsplot R package was used for graphical visualizations of the results.^23^ We used the STROBE cohort checklist when writing our report.^24^ The data that support the findings of this study are available from the corresponding author on reasonable request, within the privacy legislation of the Netherlands and after permission of the Heart-Brain Connection steering committee.

## Results

### Population characteristics

A total of 428 participants were included in this study (Supplemental Figure S1). Participants were on average 67.2 ± 8.6 years old, 37% was female, 28% of the participants had a diagnosis of heart failure, 17% of COD, 29% of VCI, and 26% were from the reference group. Of all patients, 330 had hypertension (80%), 71 were current smokers (17%) (Table 1). Participants that were excluded because of ASL-MRI images with suboptimal quality (i.e., motion artefacts, incomplete ASL-sequence, or labeling errors) and images with dominant vascular artefacts and little tissue perfusion signal, were significantly older (median age 70; IQR 64–76) than included participants (median age 67; IQR 61, 73) (p = 0.002), belonged more often to COD group (29%) than included participants (17%), and more often had hypertension (88%) compared to included participants (80%) (p = 0.04).

**Table 1.** Baseline characteristics.

	HF, N = 119^a^	COD, N = 74^a^	VCI, N = 122^a^	Reference, N = 113^a^
Sociodemographics				
Age	69 (62, 76)	66 (59, 71)	69 (61, 74)	67 (61, 70)
Female sex	37 (31%)	20 (27%)	48 (39%)	55 (49%)
Years of education	12.0 (10.0, 16.0)	12.0 (10.0, 16.0)	13.0 (10.0, 16.0)	14.0 (11.0, 17.0)
Cardiovascular risk factors				
Hypertension	103 (90%)	67 (91%)	96 (83%)	64 (59%)
Total cholesterol (mmol/l)	4.80 (4.00, 5.40)	4.70 (3.80, 5.40)	4.75 (3.98, 5.40)	5.70 (5.10, 6.20)
Diabetes	13 (11%)	10 (14%)	4 (3.3%)	1 (0.9%)
Current smoker	21 (18%)	19 (26%)	24 (20%)	7 (6.2%)
History of stroke	6 (5.0%)	38 (51%)	51 (42%)	0 (0%)
History of myocardial infarction	74 (62%)	21 (28%)	20 (16%)	7 (6.2%)
Obesity	27 (23%)	21 (28%)	19 (16%)	18 (16%)
Cardiovascular risk				
SCORE	9 (5, 15)	8 (4, 11)	8 (4, 14)	7 (4, 10)
Cerebral blood flow				
CBF (mean mL/100 g/min)	52 (45, 60)	46 (42, 55)	50 (44, 57)	56 (49, 63)

a Median (IQR); n (%).

HF: heart failure; COD: carotid occlusive disease; VCI: vascular cognitive impairment; SCORE: Systematic COronary Risk Evaluation.

### CBF is lower and cardiovascular risk is higher in cardiovascular disease patients compared with reference participants

**Figure 1.**
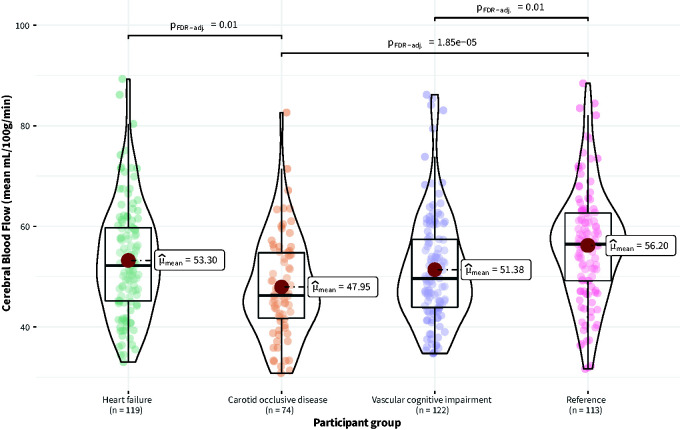
Distribution of cerebral blood flow (CBF) across participant groups (Heart failure, Carotid occlusive disease, Vascular cognitive impairment, Reference).

Mean CBF across the groups was 52 mL/100g/min (SD = 11). There was an overall statistically significant effect of participant group on CBF (F_Welch_(3, 221.76) = 9.64, p < 0.001). Mean CBF was significantly higher in the reference group compared to the VCI group (p_FDR-corrected_ = 0.01) and COD group (p_FDR-corrected_ < 0.001) (Figure 1). There was no statistically significant difference between the reference group and the HF group.

There was also an overall statistically significant effect of participant group on cardiovascular risk (SCORE) (F_Welch_(3, 219.85) = 6.95, p < 0.001). Cardiovascular risk (SCORE) was significantly lower in the reference group compared with the HF group (p_FDR-corrected_ < 0.01) and VCI group (p_FDR-corrected_ = 0.04). There was no statistically significant difference in cardiovascular risk (SCORE) between the reference group and the COD group. Across all participant groups, CBF was inversely associated with cardiovascular risk (SCORE) (ρ_Spearman_ = −0.26, CI_95%_ [−0.35, −0.17], p < 0.001) (Supplemental Figure S2).

### A protein biomarker cluster is associated with CBF

In total, eighteen of the ninety-two plasma protein biomarkers from the panel individually correlated with CBF (Table 2, Supplemental Figure S1). Fifteen of the eighteen protein biomarkers negatively correlated with CBF (ρ_Spearman_ range: −0.10 to −0.21, p_FDR-corrected_ < 0.05, Table 2). Three of the eighteen protein biomarkers positively correlated with CBF (ρ_Spearman_ range 0.12 - 0.17.; p_FDR-corrected_ < 0.05, Table 2).

**Table 2.** Overview of the 18 significantly correlating biomarkers with cerebral blood flow.

Biomarker (abbreviation)	ρ_Spearman_^a^	p-value	p_FDR-corrected_^b^
Negative association			
** **Plasminogen activator, tissue type (PLAT)	−0.211	<0.001	<0.001
** **Interleukin 1 receptor like 1 (IL1R1)	−0.163	<0.001	0.004
** **Growth differentiation factor 15 (GDF15)	−0.146	0.002	0.012
** **Peptidase inhibitor 3 (PI3)	−0.142	0.003	0.013
** **Selectin E (SELE)	−0.132	0.006	0.016
** **Cathepsin D (CTSD)	−0.129	0.007	0.016
** **Azurocidin 1 (AZU1)	−0.128	0.008	0.016
** **Matrix metallopeptidase 3 (MMP3)	−0.122	0.012	0.017
** **Heparan sulfate proteoglycan 2 (HSPG2)	−0.122	0.013	0.017
** **Matrix metallopeptidase 9 (MMP9)	−0.115	0.017	0.025
** **Cystatin B (CSTB)	−0.113	0.019	0.025
** **Insulin like growth factor binding protein 7 (IGFBP7)	−0.113	0.020	0.025
** **Acid phosphatase 5, tartrate resistant (ACP5)	−0.110	0.023	0.027
** **Myoglobin (MB)	−0.101	0.037	0.039
** **Platelet and endothelial cell adhesion molecule 1 (PECAM1)	−0.096	0.048	0.049
Positive association			
** **Secretoglobin family 3 A member 2 (SCGB3A2)	0.124	0.010	0.018
** **Contactin 1 (CNTN1)	0.139	0.030	0.033
** **Paraoxonase 3 (PON3)	0.171	<0.001	0.003

a Spearman’s rank correlation coefficient

b False Discovery Rate-corrected p-values (Benjamini & Hochberg)

**Figure 2.**
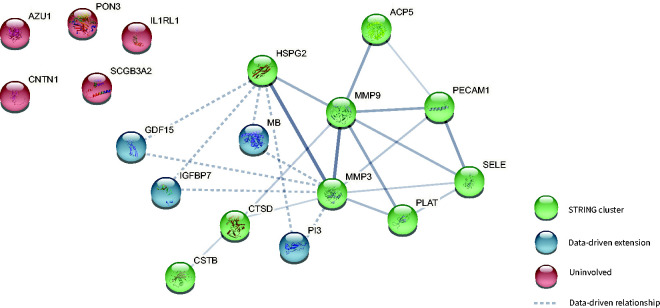
Thirteen of the 18 biomarkers that each significantly correlated with CBF formed one cluster according to both prior knowledge and data-driven cluster analyses. Biomarkers that were found to be involved based on prior knowledge (with information from the STRING database) are shown in green. Four additional biomarkers that were found to extend this cluster based on data-driven cluster analyses are shown in blue. Line width reflects the strength of data support. Data-driven extensions are shown as dashed lines.

To determine whether these biomarkers are part of similar pathways, we used cluster analyses using prior-knowledge and data-driven analyses. Plasma levels of nine of the eighteen protein biomarkers, i.e. Heparan Sulfate Proteoglycan 2 (HSPG2); Selectin E (SELE); Plasminogen activator, tissue type (PLAT); Matrix metallopeptidase 9 (MMP9); Cathepsin D (CTSD); Acid phosphatase 5, tartrare resistant (ACP5); Matrix metallopeptidase 3 (MMP3); Platelet endothelial cell adhesion molecule 1 (PECAM1); and Cystatin-B (CSTB), were found to be interrelated according to prior knowledge captured in the STRING database (protein-protein interaction p-value < 0.001) and formed one 9-component cluster (Figure 2).

Levels of another four out of the eighteen biomarkers, i.e. Myoglobin (MB); Growth differentiation factor 15 (GDF15); Peptidase inhibitor 3 (PI3) and Insulin-like growth factor-binding protein 7 (IGFBP7) were not directly interrelated with other biomarkers of the eighteen according to prior-knowledge (STRING) (Figure 2). However, using data-driven cluster analyses based on the Olink® NPX values, we observed significant associations (p < 0.05) between these four biomarkers and the previously mentioned 9-component cluster, that was interrelated based on prior knowledge, therefore, we added these four biomarkers to the 9-component cluster, which resulted in a 13-component cluster related to CBF (Figure 2).

Although the remaining five of the eighteen biomarkers, i.e., Interleukin receptor like 1 (IL1RL1), Azurocidin 1 (AZU1), Paraoxonase 3 (PON3), Contactin 1 (CNTN1), Secretoglobin family 3 A member 2 (SCGB3A2) were significantly associated with CBF, we found no relationships with respect to other protein biomarkers (Figure 2), and therefore excluded them from further analyses.

Within the 13-component cluster related to CBF, we identified 15 significantly enriched pathways (p_FDR-corrected_ < 0.05), pointing mainly towards inflammation, extracellular matrix organization, and signal transduction processes (Table 3).

**Table 3.** Statistically enriched biological pathways reflected by the identified 13-component biomarker cluster based on the whole genome.

Pathway	# Biomarkers in cluster/Total # of biomarkers in pathway (Reactome)	p	p_FDR-corrected_^a^	Biomarkers
*Inflammation pathways*				
Interleukin-4 and Interleukin-13 signaling	2/211	<0.001	<0.001	MMP3, MMP3
Neutrophil degranulation	4/480	<0.001	0.008	MMP9, CTSD, CSTB, PECAM1
Senescence-Associated Secretory Phenotype	1/91	0.005	0.038	IGFBP7
*Extracellular matrix organization pathways*				
Degradation of the extracellular matrix	4/148	<0.001	0.001	MMP9, CTSD, MMP3, HSPG2
Extracellular matrix organization	5/301	<0.001	0.001	MMP9, CTSD, PECAM1, MMP3, HSPG2
Collagen degradation	3/64	<0.001	0.002	MMP9, CTSD, MMP3
Activation of Matrix Metalloproteinases	2/33	0.001	0.015	MMP9, MMP3
Assembly of collagen fibrils and other multimeric structures	2/61	0.003	0.030	MMP9, MMP3
Collagen formation	2/90	0.007	0.036	MMP9, MMP3
Integrin cell surface interactions	2/85	0.006	0.036	PECAM1, HSPG2
*Signal transduction pathways*				
ESR-mediated signaling	3/196	0.003	0.028	MMP9, CTSD, MMP3
Signaling by Interleukins	4/457	0.003	0.030	MMP9, MMP3
Signaling by Nuclear Receptors	3/273	0.006	0.036	MMP9, CTSD, MMP3
Extra-nuclear estrogen signaling	2/80	0.006	0.036	MMP9, MMP3
*Transport of small molecules pathway*				
Intracellular oxygen transport	1/5	0.006	0.038	MB

a False Discovery Rate-corrected p-values

The number of biomarkers involved in the cluster and belonging to the pathway are shown, as a ratio to the known total number of biomarkers involved in the pathway based on the Reactome pathway analysis tool. For example, 480 proteins are known to be involved in Neutrophil degranulation, of which four are part of our 13-component cluster (MMP9, CTSD, CSTB, PECAM1), this pathway is significantly enriched (p_FDR-corrected_ < 0.001).

The BCS showed a significant negative association with CBF (ρ_Spearman_ = −0.25, p < 0.001) (Supplemental Figure S2), a stronger association than all the individual associations between the biomarkers and CBF. Similar results were seen for the phase contrast flow measures (ρ_Spearman_ = −0.12, CI_95%_ [−0.21, −0.02], p = 0.02). After stratification by participant group, we identified a negative correlation between the BCS and CBF within each of the participant groups (Supplemental Figure S3).

### The protein biomarker cluster is associated with cardiovascular risk and events

**Figure 3.**
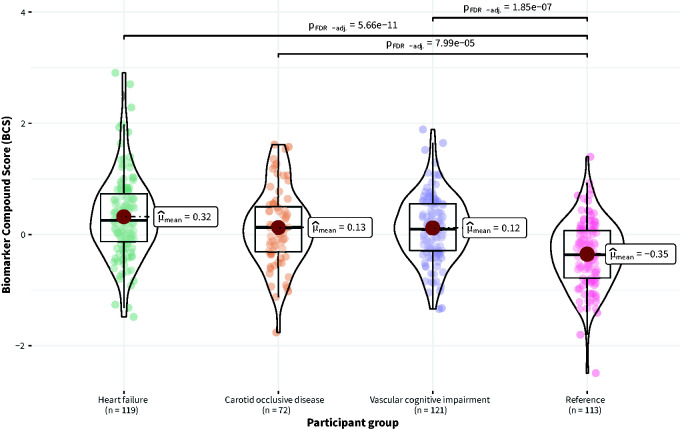
Distribution of the Biomarker Compound Score (BCS) in Normalized Protein eXpression (NPX) across participant groups (Heart failure, Carotid occlusive disease, Vascular cognitive impairment, Reference).

There was an overall statistically significant effect of participant group on the BCS (F_Welch_(3, 212.84) = 22.13, p < 0.001). The BCS was significantly lower in the reference group compared to the VCI group (p_FDR-corrected_ < 0.001), HF group (p_FDR-corrected_ < 0.001), and COD group (p_FDR-corrected_ < 0.001) (Figure 3).

Moreover, the BCS was significantly and positively associated with cardiovascular risk (SCORE) (ρ_Spearman_ −0.42, p < 0.001) (Supplemental Figure S4). In total, 63 cardiovascular events in 428 participants were observed during two-year follow-up (Supplemental Table S2). We observed a nonsignificant trend between CBF and cardiovascular events (OR = 0.80, CI_95% _=−0.71, 1.10, p-value = 0.20). However, across all participants, the BCS was significantly associated with events when corrected for cardiovascular risk (SCORE) (log(OR) per SD increase in BCS = 0.78, CI_95%_ [0.28, 1.3], p < 0.01) (Supplemental Table S3).

## Discussion

Using data of participants with different hemodynamic disturbances and a reference group, we identified thirteen protein biomarkers related to decreased CBF. These biomarkers formed a cluster based on a combination of prior knowledge on protein-protein interactions and data-driven clustering algorithms. This cluster of biomarkers reflects inflammation, extracellular matrix organization, and signal transduction processes. The biomarker compound score (BCS), calculated based on the biomarkers involved in the cluster, is associated with cardiovascular risk. In addition, the BCS was related to future cardiovascular events, independent of traditional cardiovascular risk factors.

In this study we assessed the relation between a large panel of protein biomarkers and decreased CBF in the context of CVD. In previous studies, single proteins that are expected to have a relationship with the outcome were usually selected and examined univariately. In one such study, a relationship between a marker of endothelial dysfunction and decreased CBF was demonstrated.^25^ With such a unimodal approach, it is possible to pick up a signal in individual protein biomarkers, but it does not capture the network nature that more closely resembles biology.

The method we used previously proved effective in research on CVD, where we expanded upon prior knowledge on protein-protein interactions to assure biological relevance.^7^ Since not all existing protein-protein interactions are already known, we used both prior knowledge and data-driven cluster analyses based on our Olink® data to suggest novel protein-protein interactions. This combination of prior knowledge and data-driven cluster analyses also proved effective in previous studies, such as in cerebral small vessel disease,^7^ diabetes,^26^ heart failure,^27,28^ and cancer.^6,29^

Of note, the mean differences in CBF between patients and controls were modest and the relationship between lower CBF and MACE was not statistically significant. Yet, this is to be expected for a physiological measure like CBF. Due to biological homeostatic drive fundamental physiological features like CBF do not become markedly abnormal even in disease states and do not fully separate patients from controls, as we see in our results, despite the CBF in the patient groups being significantly lower than controls. Nevertheless, interindividual variation in CBF may reflect an important biological signal, as the current findings also seem to imply. Pathway analysis showed that the identified biomarker network predominantly reflects inflammation, extracellular matrix organization, and signal transduction processes. These findings are in line with experimental studies that found a relation between cerebral hypoperfusion and both inflammation and extracellular matrix organization processes.^3,30–32^ Although the design of this study precludes us from establishing causality, our study extends previous findings by indicating that inflammation, extracellular matrix organization and signal transduction processes appear to be related to decreased cerebral blood flow in humans. Such relations may be bidirectional. Alterations in protein biomarkers may reflect processes that contribute to lower CBF and brain injury. Conversely, lower CBF itself may induce changes in the levels of certain markers. Based on rodent models it is hypothesized that cerebral hypoperfusion is associated with several downstream events,^32^ including activation of inflammatory responses and induction of signal transductions pathways, which in turn may lead to production of MMPs that open the blood brain barrier by disrupting tight junctions and extracellular matrix. Disruption of the blood brain barrier is considered to cause vasogenic edema which subsequently may accelerate brain tissue damage and vice versa may lead to cerebral hypoperfusion. On the other hand, the biological processes identified with the biomarker cluster may reflect certain upstream events that may cause reduced CBF. For example, systemic inflammation may impair the normal hemodynamic regulation of cerebral vessels due to various factors, including endothelial dysfunction.^33^ Studying causality between the identified biological processes and reduced CBF in future studies is important for considering these biological processes as targets for prevention and treatment of cerebral hypoperfusion.

Strengths of this study are the comprehensive cluster analyses that enabled us to pick up a network that may be involved in decreased CBF. The BCS based on interrelated plasma proteins showed a stronger association with decreased CBF compared to when all biomarkers were analyzed univariately, indicating that the compound score performs better than the sum of its parts. In addition, this comprehensive, data-driven method to compute a BCS is most likely generalizable to other studies involving multimarker protein panels. Next, the identified cluster and related pathways could potentially be validated with other proteomics software such as Ingenuity Pathway Analysis (IPA).^34^ For the current research we preferred STRING’s publicly available software in this study as opposed to a commercial platform like IPA, also because of STRING’s visualization capabilities. Additionally, we studied a cohort of patients with three different types of CVD comprising different components of the heart-brain axis, and found similar results, which makes our results generalizable.

Several limitations should be considered. First, a form of selection bias was present because we excluded participants with missing pCASL or pCASL with suboptimal quality or vascular artefacts. In addition, the interpretation of CBF measures with pCASL is more difficult in patients with CVD because of prolonged arterial transit times. However, we performed a sensitivity analysis by calculating the association between the BCS and phase contrast flow measures and found similar results, indicating that the limitations did not have a major impact on the results. Second, the differences in CBF between the groups were relatively subtle and still largely within the normal range. However, it is conceivable that even long-term subtle changes in CBF, and the processes associated with it, are unfavorable for the brain. Third, although additional analysis within participant groups showed that our findings hold true for different hemodynamic disturbances and the reference group, we did not have enough statistical power to perform the cluster analysis separate per participant group. Also, this study only includes participants who live in the Netherlands, which may limit the generalizability of our findings to patients from other demographic regions. Additionally, a subset of 92 proteins of all proteins in the circulation was selected based on their relationship with CVD, which may have given rise to selection bias. However, of all the 92 proteins from the panel, only 18 proteins were associated with reduced CBF, the other 74 proteins were not, which suggests that within the preselected panel, subprocesses might be related to reduced CBF. Using the subcluster, we performed a pathway analysis relative to the entire proteome to provide biological meaning. We are aware that the panel pre-selection makes the traditional methods of calculating biological enrichment suboptimal for our research purposes. Larger protein panels without strong pre-selection are required to achieve results where selection bias plays a less significant role. We hope that with this addition we have adequately touched upon this point. Furthermore, although we performed internal validation by using bootstrapping in the cluster analysis, future studies with alternative and larger biomarker panels should be performed to validate and extend our findings. Additionally, in our current study, we tried to identify associations within a subset of biomarkers. We assessed direct interaction of the proteins of interest with each other based on the information from the STRING database without including further proteins from the respective biological pathway, as these were not included in the targeted proteomics approach. The identified cluster is possibly part of a larger cluster that may be involved in reduced CBF which should be considered in follow-up studies. Lastly, regarding clinical outcome, we limited ourselves to major adverse cardiovascular events for proof of concept of clinical relevance; future studies may further explore the role of the BCS for other clinical outcomes, such as loss of brain health by measuring neurovascular coupling, vasodilator capacity, and collateral vessel function, but this was beyond the scope of the current study.

We imply that clustering methods are helpful in finding biological processes involved in decreased CBF and suggest a role for specific processes involved in inflammation, extracellular matrix organization, and signal transduction. These insights can be used to better understand the biological processes involved in decreased CBF. In future studies, it would be interesting to analyze larger biomarker panels, including both plasma and cerebrospinal fluid biomarkers, by using proteomics to reduce selection bias and be able to pick up larger biomarker clusters to further unravel the pathophysiology of decreased CBF. Importantly, we also demonstrated an association between the protein biomarker cluster and both cardiovascular risk and occurrence of cardiovascular events within two years. Interestingly, the latter was independent of cardiovascular risk. If our results are validated in future studies, the protein clusters may help for further risk stratification.

In conclusion, we identified a cluster of blood-based biomarkers related to reduced CBF in patients with CVD. The biomarker cluster was related to cardiovascular risk and future cardiovascular events in patients with CVD. The plasma proteins included in the cluster reflect extracellular matrix organization, inflammation, and signal transduction processes, suggesting the involvement of these biological processes in the pathophysiology of reduced CBF. If validated in future studies, these biological processes might be therapeutic targets for preserving CBF, reduce risk of cardiovascular events and improving cognitive outcomes in people with CVD.

## Supplemental Material

sj-pdf-1-jcb-10.1177_0271678X231195243 - Supplemental material for A cluster of blood-based protein biomarkers associated with decreased cerebral blood flow relates to future cardiovascular events in patients with cardiovascular disease Supplemental material, sj-pdf-1-jcb-10.1177_0271678X231195243 for A cluster of blood-based protein biomarkers associated with decreased cerebral blood flow relates to future cardiovascular events in patients with cardiovascular disease by L Malin Overmars, Sanne Kuipers, Bram van Es, Jeroen de Bresser, Esther E Bron, Imo E Hoefer, Wouter W Van Solinge, L Jaap Kappelle, Matthias JP van Osch, Charlotte E Teunissen, Geert Jan Biessels, Saskia Haitjema and Heart-Brain Connection Consortium in Journal of Cerebral Blood Flow & Metabolism

## References

[1] Abete P Della-Morte D Gargiulo G , et al. Cognitive impairment and cardiovascular diseases in the elderly. A heart-brain continuum hypothesis. Ageing Res Rev 2014; 18: 41–52. 25107566 10.1016/j.arr.2014.07.003

[2] Hooghiemstra AM Bertens AS Leeuwis AE , et al. The missing link in the pathophysiology of vascular cognitive impairment: design of the heart-brain study. Cerebrovasc Dis Extra 2017; 7: 140–152. 29017156 10.1159/000480738 PMC5730112

[3] Iadecola C. The pathobiology of vascular dementia. Neuron 2013; 80: 844–866. 24267647 10.1016/j.neuron.2013.10.008 PMC3842016

[4] Ihara M Tomimoto H Kinoshita M , et al. Chronic cerebral hypoperfusion induces MMP-2 but not MMP-9 expression in the microglia and vascular endothelium of white matter. J Cereb Blood Flow Metab 2001; 21: 828–834. 11435795 10.1097/00004647-200107000-00008

[5] Altendahl M Maillard P Harvey D , et al. An IL-18-centered inflammatory network as a biomarker for cerebral white matter injury. PLoS One 2020; 15: e0227835. 31978079 10.1371/journal.pone.0227835 PMC6980497

[bibr6-0271678X231195243] Luo T Wu S Shen X , et al. Network cluster analysis of protein-protein interaction network identified biomarker for early onset colorectal cancer. Mol Biol Rep 2013; 40: 6561–6568. 24197691 10.1007/s11033-013-2694-0

[7] Kuipers S Overmars LM van Es B , et al. A cluster of blood-based protein biomarkers reflecting coagulation relates to the burden of cerebral small vessel disease. J Cereb Blood Flow Metab 2022; 42: 1282–1293. 35086368 10.1177/0271678X221077339 PMC9207498

[8] Olink P. Olink target Cardiovascular III, www.olink.com/products-services/target/cardiovascular-iii-panel/ (accessed 8 February 2022).

[9] Lind L Wallentin L Kempf T , et al. Growth-differentiation factor-15 is an independent marker of cardiovascular dysfunction and disease in the elderly: results from the prospective investigation of the vasculature in Uppsala seniors (PIVUS) study. Eur Heart J 2009; 30: 2346–2353. 19561023 10.1093/eurheartj/ehp261

[bibr10-0271678X231195243] Wollert KC Kempf T Peter T , et al. Prognostic value of growth-differentiation factor-15 in patients with non-ST-elevation acute coronary syndrome. Circulation 2007; 115: 962–971. 17283261 10.1161/CIRCULATIONAHA.106.650846

[11] Shah RV Januzzi JL. ST2: a novel remodeling biomarker in acute and chronic heart failure. Curr Heart Fail Rep 2010; 7: 9–14. 20425491 10.1007/s11897-010-0005-9

[12] Alsop DC Detre JA Golay X , et al. Recommended implementation of arterial spin-labeled perfusion MRI for clinical applications: a consensus of the ISMRM perfusion study group and the European Consortium for ASL in Dementia. Magn Reson Med 2015; 73: 102–116. 24715426 10.1002/mrm.25197 PMC4190138

[13] Bron EE Steketee RME Houston GC , et al. Diagnostic classification of arterial spin labeling and structural MRI in presenile early stage dementia. Hum Brain Mapp 2014; 35: 4916–4931. 24700485 10.1002/hbm.22522 PMC6869162

[14] Huizinga W Poot DHJ Guyader J-M , et al. PCA-based groupwise image registration for quantitative MRI. Med Image Anal 2016; 29: 65–78. 26802910 10.1016/j.media.2015.12.004

[15] Leeuwis AE Hooghiemstra AM Bron EE , et al. Cerebral blood flow and cognitive functioning in patients with disorders along the heart-brain axis: cerebral blood flow and the heart-brain axis. Alzheimers Dement (N Y) 2020; 6: e12034. 32995468 10.1002/trc2.12034 PMC7507476

[16] Oudeman EA Bron EE Van den Berg-Vos RM , et al. Cerebral perfusion and the occurrence of nonfocal transient neurological attacks. Cerebrovasc Dis 2019; 47: 303–308. 31422397 10.1159/000502334

[17] Conroy RM Pyörälä K Fitzgerald AP , et al. Estimation of ten-year risk of fatal cardiovascular disease in Europe: the SCORE project. Eur Heart J 2003; 24: 987–1003. 12788299 10.1016/s0195-668x(03)00114-3

[18] Szklarczyk D Gable AL Lyon D , et al. STRING v11: protein-protein association networks with increased coverage, supporting functional discovery in genome-wide experimental datasets. Nucleic Acids Res 2019; 47: D607–D613. 30476243 10.1093/nar/gky1131 PMC6323986

[19] Frey BJ Dueck D. Clustering by passing messages between data points. Science 2007; 315: 972–976. 17218491 10.1126/science.1136800

[20] Jassal B Matthews L Viteri G , et al. The reactome pathway knowledgebase. Nucleic Acids Res 2020; 48: D498–D503. 31691815 10.1093/nar/gkz1031 PMC7145712

[21] Storn R Price K. Differential evolution – a simple and efficient heuristic for global optimization over continuous spaces. J Global Optimiz 1997; 11: 341–359.

[22] Virtanen P Gommers R Oliphant TE , et al. SciPy 1.0: fundamental algorithms for scientific computing in python. Nat Methods 2020; 17: 261–272. 32015543 10.1038/s41592-019-0686-2 PMC7056644

[23] Patil I. Visualizations with statistical details: the “ggstatsplot” approach. JOSS 2021; 6: 3167.

[24] von Elm E Altman DG Egger M , et al. The strengthening the reporting of observational studies in epidemiology (STROBE) statement: guidelines for reporting observational studies. J Clin Epidemiol 2008; 61: 344–349. 18313558 10.1016/j.jclinepi.2007.11.008

[25] Tchalla AE Wellenius GA Travison TG , et al. Circulating vascular cell adhesion molecule-1 is associated with cerebral blood flow dysregulation, mobility impairment, and falls in older adults. Hypertension 2015; 66: 340–346. 26056332 10.1161/HYPERTENSIONAHA.115.05180 PMC4807019

[26] Li Z Qiao Z Zheng W , et al. Network cluster analysis of protein-protein interaction network-identified biomarker for type 2 diabetes. Diabetes Technol Ther 2015; 17: 475–481. 25879401 10.1089/dia.2014.0204 PMC4504429

[27] Sanders-van Wijk S Tromp J Beussink-Nelson L , et al. Proteomic evaluation of the comorbidity-inflammation paradigm in heart failure with preserved ejection fraction: results from the PROMIS-HFpEF study. Circulation 2020; 142: 2029–2044. 33034202 10.1161/CIRCULATIONAHA.120.045810 PMC7686082

[28] Sama IE Woolley RJ Nauta JF , et al. A network analysis to identify pathophysiological pathways distinguishing ischaemic from non-ischaemic heart failure. Eur J Heart Fail 2020; 22: 821–833. 32243695 10.1002/ejhf.1811 PMC7319432

[29] Hu K Chen F. Identification of significant pathways in gastric cancer based on protein-protein interaction networks and cluster analysis. Genet Mol Biol 2012; 35: 701–708. 23055812 10.1590/S1415-47572012005000045 PMC3459423

[30] Sood R Yang Y Taheri S , et al. Increased apparent diffusion coefficients on MRI linked with matrix metalloproteinases and edema in white matter after bilateral carotid artery occlusion in rats. J Cereb Blood Flow Metab 2009; 29: 308–316. 18941468 10.1038/jcbfm.2008.121

[bibr31-0271678X231195243] Nakaji K Ihara M Takahashi C , et al. Matrix metalloproteinase-2 plays a critical role in the pathogenesis of white matter lesions after chronic cerebral hypoperfusion in rodents. Stroke 2006; 37: 2816–2823. 17008622 10.1161/01.STR.0000244808.17972.55

[32] Jalal FY Yang Y Thompson JF , et al. Hypoxia-induced neuroinflammatory white-matter injury reduced by minocycline in SHR/SP. J Cereb Blood Flow Metab 2015; 35: 1145–1153. 25712499 10.1038/jcbfm.2015.21 PMC4640265

[33] Sabayan B Westendorp RG van der Grond J , et al. Markers of endothelial dysfunction and cerebral blood flow in older adults. Neurobiol Aging 2014; 35: 373–377. 24054993 10.1016/j.neurobiolaging.2013.08.020

[34] Müller T Schrötter A Loosse C , et al. Sense and nonsense of pathway analysis software in proteomics. J Proteome Res 2011; 10: 5398–5408. 21978018 10.1021/pr200654k

