# Supplementary material for: A cluster of blood-based protein biomarkers associated with decreased cerebral blood flow relates to future cardiovascular events in patients with cardiovascular disease
Source: J Cereb Blood Flow Metab. 2023 Aug 12;43(12):2060–71. doi: 10.1177/0271678X231195243 (PMC10925867; doi:10.1177/0271678X231195243)
Supplement: sj-pdf-1-jcb-10.1177_0271678X231195243 - Supplemental material for A cluster of blood-based protein biomarkers associated with decreased cerebral blood flow relates to future cardiovascular events in patients with cardiovascular disease [file sj-pdf-1-jcb-10.1177_0271678X231195243.pdf]

Supplemental material

Supplemental Table S1. List of 92 biomarkers involved in the OLINK cardiovascular III panel and medians with interquartile ranges

Protein	N = 428[1]
TNFRSF14	4.10 (3.87, 4.38)
LDLreceptor	3.30 (2.95, 3.74)
ITGB2	4.55 (4.29, 4.78)
IL17RA	3.60 (3.20, 3.93)
TNFR2	5.01 (4.71, 5.31)
MMP9	4.45 (3.81, 4.98)
EPHB4	4.94 (4.74, 5.15)
IL2RA	3.34 (3.04, 3.63)
OPG	3.40 (3.18, 3.58)
ALCAM	6.99 (6.85, 7.21)
TFF3	4.80 (4.59, 5.14)
SELP	9.37 (9.02, 9.73)
CSTB	3.51 (3.19, 3.91)
MCP1	3.42 (3.20, 3.74)
CD163	7.28 (6.94, 7.63)

Protein	N = 428[1]
Gal3	2.64 (2.40, 2.87)
GRN	4.97 (4.81, 5.18)
BLMhydrolase	1.91 (1.72, 2.17)
PLC	7.61 (7.46, 7.84)
LTBR	3.15 (2.93, 3.38)
Notch3	4.89 (4.66, 5.14)
TIMP4	3.06 (2.72, 3.39)
CNTN1	3.95 (3.73, 4.18)
CDH5	3.54 (3.34, 3.77)
TLT2	4.56 (4.29, 4.88)
FABP4	5.15 (4.61, 5.73)
TFPI	8.68 (8.44, 8.89)
PAI	4.79 (4.30, 5.44)
CCL24	4.48 (3.87, 5.17)
TR	4.57 (4.20, 4.95)
TNFRSF10C	6.19 (5.78, 6.48)
GDF15	5.48 (5.06, 5.90)

Protein	N = 428[1]
SELE	11.46 (11.03, 11.87)
AZU1	1.46 (1.19, 1.77)
DLK1	5.36 (4.96, 5.83)
SPON1	1.23 (1.05, 1.38)
MPO	2.48 (2.23, 2.71)
CXCL16	4.74 (4.56, 4.90)
IL6RA	11.53 (11.23, 11.78)
RETN	5.79 (5.48, 6.17)
IGFBP1	3.63 (2.80, 4.40)
CHIT1	4.92 (4.16, 5.53)
TRAP	3.06 (2.80, 3.30)
GP6	2.21 (1.56, 2.73)
PSPD	2.36 (1.93, 2.82)
PI3	1.72 (1.33, 2.17)
EpCAM	4.97 (4.28, 5.77)
APN	4.34 (4.19, 4.51)
AXL	8.19 (7.99, 8.41)

Protein	N = 428[1]
IL1RT1	5.78 (5.61, 5.98)
MMP2	3.14 (2.93, 3.37)
FAS	5.41 (5.19, 5.62)
MB	7.07 (6.70, 7.57)
TNFSF13B	6.74 (6.52, 6.99)
PRTN3	3.11 (2.85, 3.40)
PCSK9	2.45 (2.20, 2.71)
UPAR	4.77 (4.50, 5.03)
OPN	6.75 (6.40, 7.10)
CTSD	2.01 (1.74, 2.31)
PGLYRP1	6.96 (6.62, 7.34)
CPA1	5.41 (4.94, 5.84)
JAMA	3.84 (3.47, 4.27)
Gal4	3.17 (2.83, 3.55)
IL1RT2	4.75 (4.56, 4.94)
SHPS1	3.08 (2.77, 3.33)
CCL15	6.38 (6.13, 6.82)

Protein	N = 428[1]
CASP3	6.89 (5.82, 7.59)
uPA	4.10 (3.90, 4.31)
CPB1	5.24 (4.80, 5.67)
CHI3L1	3.71 (3.14, 4.53)
ST2	3.67 (3.37, 4.00)
tPA	6.07 (5.66, 6.39)
SCGB3A2	2.08 (1.52, 2.58)
EGFR	2.46 (2.34, 2.58)
IGFBP7	7.23 (7.03, 7.45)
CD93	10.65 (10.44, 10.86)
IL18BP	5.34 (5.11, 5.59)
COL1A1	2.60 (2.39, 2.88)
PON3	5.60 (5.18, 6.05)
CTSZ	4.88 (4.62, 5.14)
MMP3	7.12 (6.58, 7.56)
RARRES2	10.90 (10.68, 11.15)
ICAM2	4.64 (4.39, 4.90)

Protein	N = 428[1]
KLK6	1.65 (1.40, 1.95)
PDGFsubunitA	2.73 (2.17, 3.32)
TNFR1	5.93 (5.70, 6.26)
IGFBP2	7.54 (7.01, 8.05)
vWF	6.88 (6.30, 7.34)
PECAM1	4.00 (3.75, 4.26)
MEPE	5.02 (4.72, 5.34)
CCL16	5.85 (5.51, 6.19)
NTproBNP	3.77 (2.93, 4.76)

[1] **Median (IQR)**

Supplemental Table S2. Frequency of different types of cardiovascular events

**Event types**	N
**Cerebrovascular events**	
Transient Ischemic Attack (TIA)	6
Ischemic stroke	5
Intracerebral hemorrhage	2
Subarachnoid hemorrhage	3

**Cardiovascular events**	
Angina pectoris	12
Myocardial infarction	6
Angioplasty/stent	7
Coronary bypass	1
Other (e.g. ICD implantation, cardioversion, ablation)	22

Supplemental Table S3. Logistic regression model results with cardiovascular events as outcome.

Variable	log(OR)[1]	95% CI[1]	p-value
**BCS**[2]	0.78	0.28, 1.3	0.003
**SCORE**[2]	-2.1	-7.8, 3.0	0.4

1 OR = Odds Ratio, CI = Confidence Interval

2 BCS = Biomarker Compound Score, SCORE = Systematic COronary Risk Evaluation

Supplemental Figure S1. Flowchart of the study population selection.

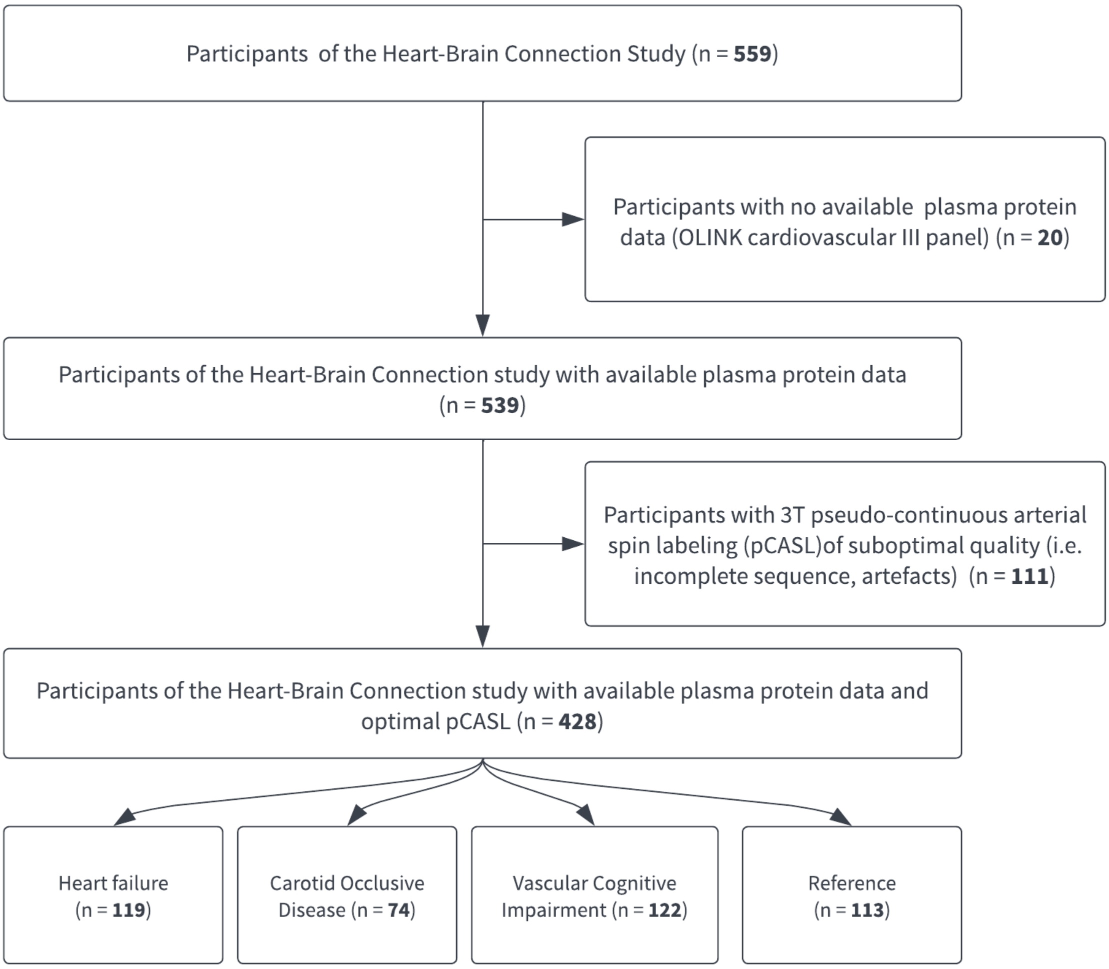

Supplemental Figure S2. Relationship between cerebral blood flow (CBF) and cardiovascular risk (SCORE)

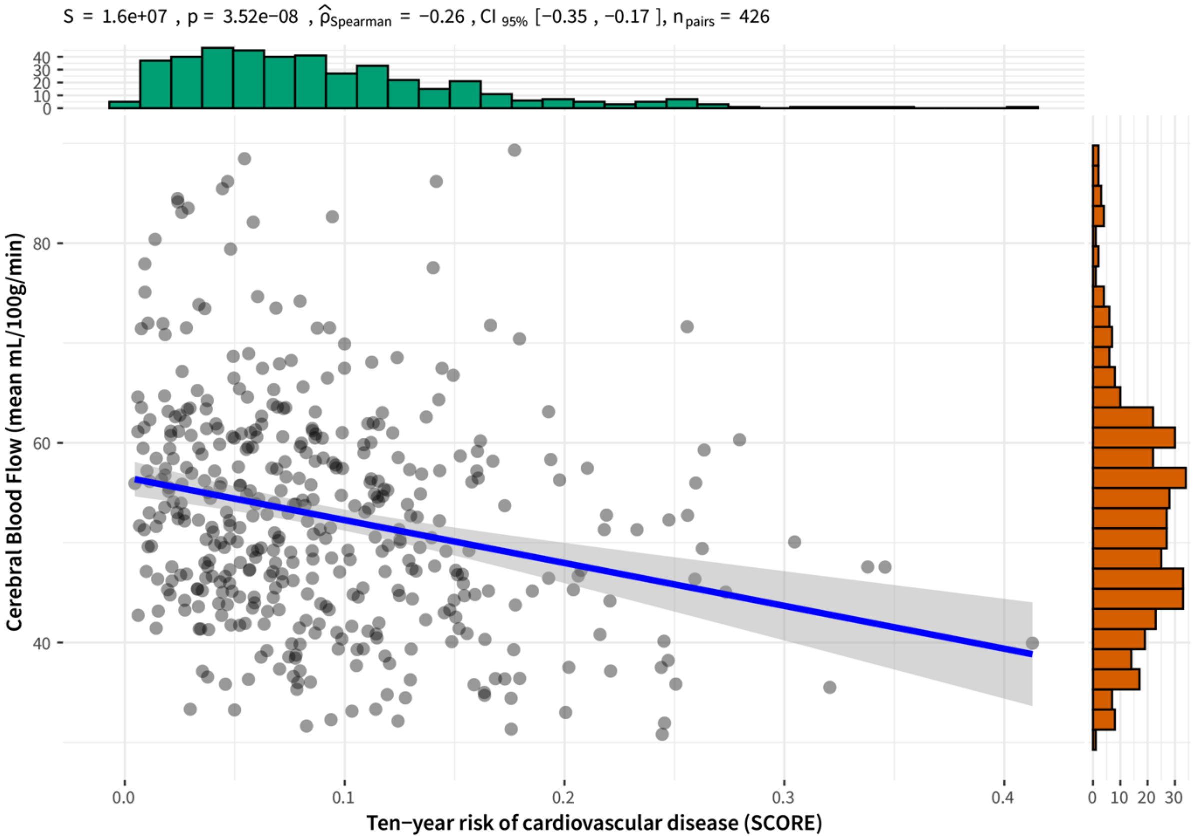

The histograms show the distribution of CBF (orange) and SCORE (green).

Supplemental Figure S3. Relationship between the Biomarker Compound Score (BCS) in NPX and Cerebral Blood Flow (CBF) in mL/100g/min, stratified by participant group

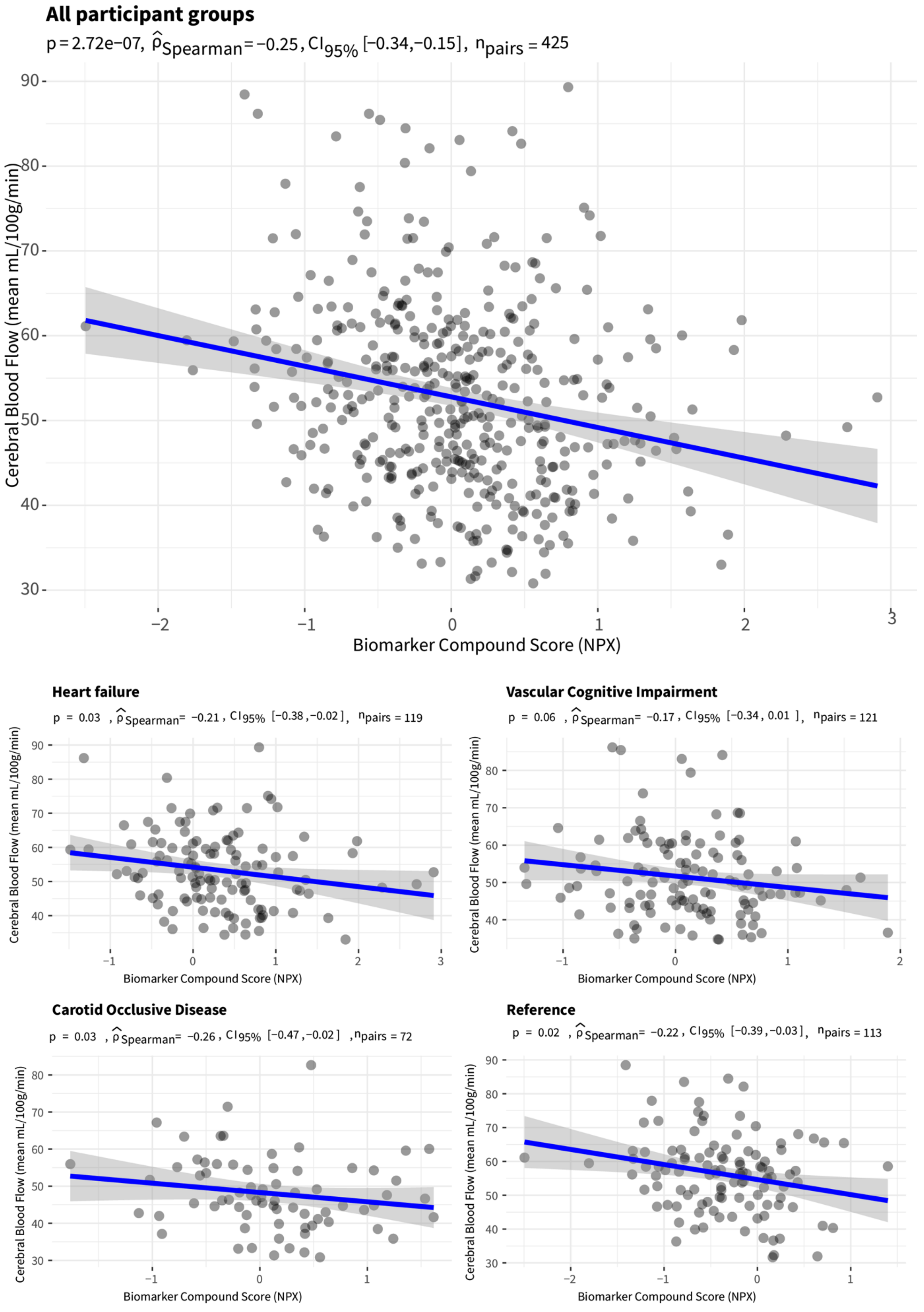

Supplemental Figure S4

Relationship between the Biomarker Compound Score (BCS) in Normalized Protein eXpression (NPX) and cardiovascular risk (SCORE).

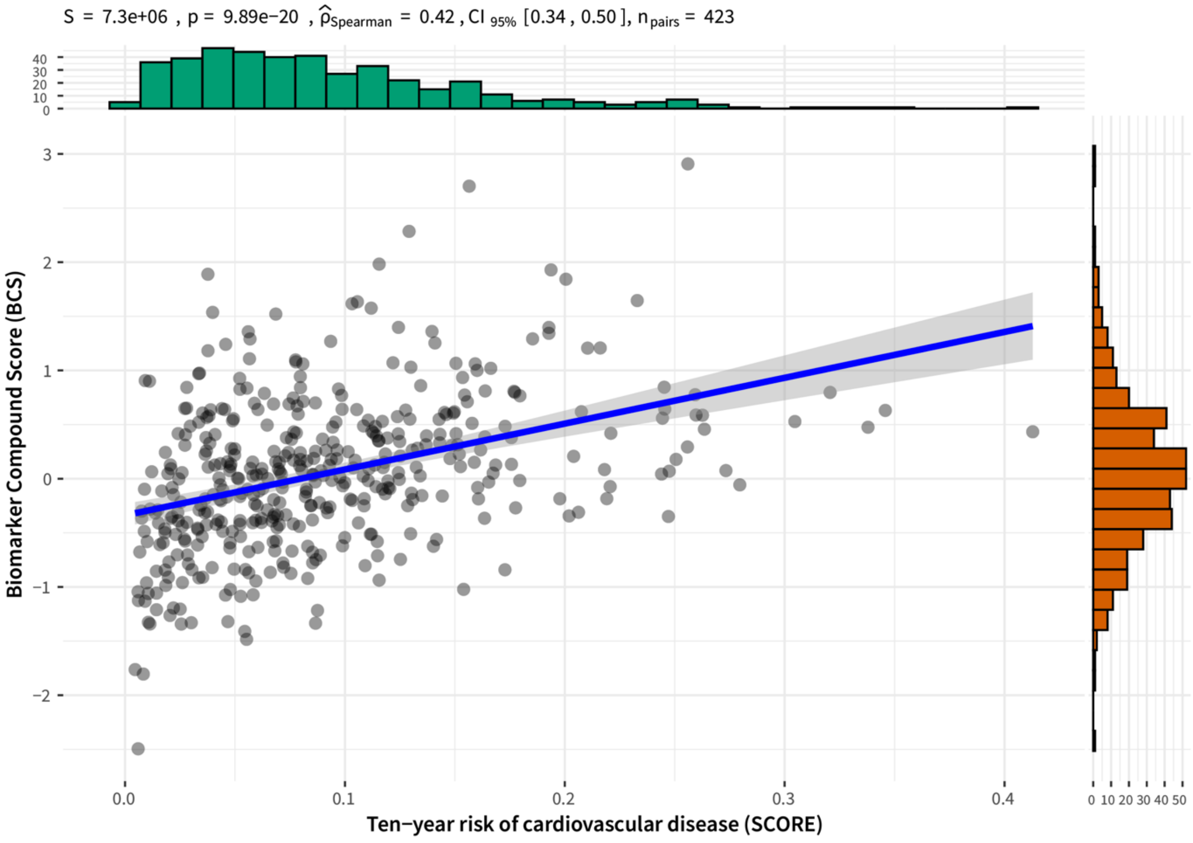

The histograms show the distribution of the BCS (orange) and SCORE (green).
